# Simple method for the generation of multiple homogeneous field volumes inside the bore of superconducting magnets

**DOI:** 10.1038/srep12200

**Published:** 2015-07-17

**Authors:** Ching-Yu Chou, Fabien Ferrage, Guy Aubert, Dimitris Sakellariou

**Affiliations:** 1 École Normale Supérieure - PSL Research University, Département de Chimie, Sorbonne Universités - UPMC Univ Paris 06, CNRS UMR 7203 LBM, 24 rue Lhomond, 75005 Paris, France; 2 CEA Saclay, DSM, IRFU, SACM, F-91191 Gif-sur-Yvette Cedex, France; 3 Laboratoire Structure et Dynamique par Résonance Magnétique, NIMBE UMR 3685 CEA/CNRS, IRAMIS, DSM, CEA Saclay, F-91191, Gif-sur-Yvette Cedex, France

## Abstract

Standard Magnetic Resonance magnets produce a single homogeneous field volume, where the analysis is performed. Nonetheless, several modern applications could benefit from the generation of multiple homogeneous field volumes along the axis and inside the bore of the magnet. In this communication, we propose a straightforward method using a combination of ring structures of permanent magnets in order to cancel the gradient of the stray field in a series of distinct volumes. These concepts were demonstrated numerically on an experimentally measured magnetic field profile. We discuss advantages and limitations of our method and present the key steps required for an experimental validation.

Modern high-field Nuclear Magnetic Resonance (NMR) is performed with superconducting magnets, which produce an axially symmetric magnetic field with a single homogeneous field volume. Usually the sample is polarized, in this highly uniform volume so-called “sweet spot”, where the field is also maximum. Spin evolution and signal detection are also performed in the same place for most conventional studies. Magnetic field uniformity of the order of parts per billion, over cm^3^ sample volumes is of prime importance for NMR spectroscopy in order to distinguish spectral features coming from very fine interactions, like the chemical shift, or the scalar couplings between nuclei. Stringent field uniformity of the order of parts per million, over dm^3^ object volumes is also important in MRI in order to obtain images having high definition. Recent NMR techniques have additionally demonstrated an increasing interest in the use of lower magnetic fields for magnetization preparation (for example pre-polarization)^1^,^2^, for spin evolution (for example relaxometry)^3^,^4^,^5^,^6^, or even for detection^7^, in combination with the standard high-field sweet spot produced by the superconducting NMR magnet.

Although making a special superconducting magnet with two homogeneous centers is possible^8^, many experimentalists might benefit from a more flexible and low-cost solution in generating multiple homogeneous spots inside the bore of their commercial superconducting magnet. Secondary homogeneous spots in the stray field of a superconducting magnet have been generated with electronic magnet, such as Helmholtz coils, outside of the Dewar^9^. In order to obtain a homogeneous spot closer to the primary high-field spot and use efficiently the stray field of the superconducting magnet, ferromagnetic inserts have been designed to compensate the gradient inside the bore of a superconducting magnet^10^. Even though ferromagnetic materials are commonly used to correct small field imperfections in high field MRI devices (a procedure called passive shimming^11^), their material properties (magnetization saturation^12^), does not facilitate large gradient compensations. Furthermore, a solution where the value of the field and the shape of the sweet-spots can be chosen by design could lead to optimized experiments for a variety of modern approaches.

In this communication, we propose a straightforward method using cylindrical rings of permanent magnet materials to eliminate the gradient of the stray field of the superconducting magnet along its center axis. Our calculations extend the range of accessible magnetic fields for the secondary homogeneous spots towards higher fields than the current design with ferromagnetic shims. In the limit of small samples, (ca. 2 mm long) our approach allows for the design of multiple volumes of homogeneous magnetic fields. Numerical calculations demonstrate the feasibility of this strategy on the experimentally measured magnetic field profile of a standard 11.7 T wide-bore superconducting magnet. We discuss future developments and limitations of our instrumentation.

## Theory/Methods

**Figure 1:**
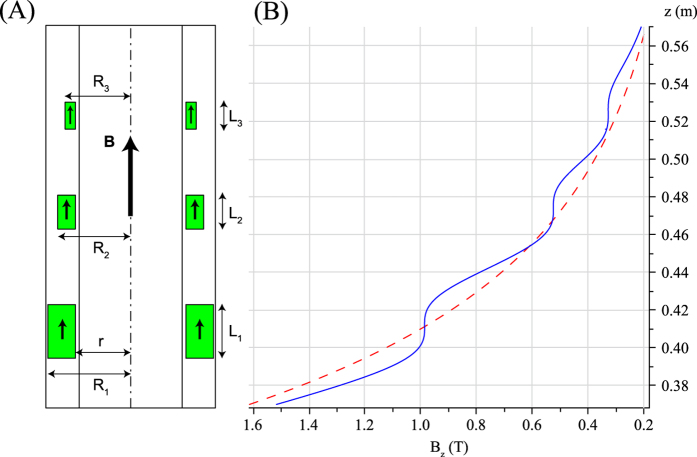
(**A**) Schematic diagram of the bore of the superconducting magnet together with three axially magnetized, rectangular cross section permanent magnet rings. The green color represents the fact that the ring magnetizations are parallel to the magnetic field of the superconducting magnet. (**B**) Magnetic field profiles without (red dashed curve) and with (blue plain curve) the compensation from the three rings. The red curve comes from the interpolation of measured data from an 11.7 T wide bore magnet, while the blue curve is calculated assuming perfectly uniform magnetization inside each ring. Here, a first order compensation was realized to provide three sweet spots in the stray field of the superconducting magnet. The *z* coordinate corresponds to the distance from the center of the superconducting magnet.

**Figure 2:**
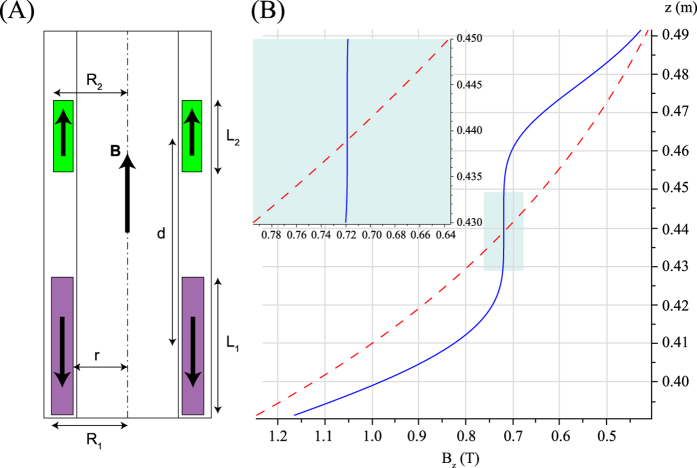
(**A**) Schematic diagram of the bore of the superconducting magnet together with two axially magnetized, rectangular cross section permanent magnet rings. The magnet rings are depicted in green (resp. magenta) if the magnetization is parallel (resp. antiparallel) to the magnetic field of the superconducting magnet. (**B**) Magnetic field profiles without (red dashed curve) and with (blue plain curve) the compensation from the two rings. The red curve comes from the interpolation of measured data from an 11.7 T wide bore magnet, while the blue curve is calculated assuming perfectly uniform magnetization inside each ring. Here a third order compensation was realized to provide a much larger sweet spot as can be appreciated in the insert of (B). The *z* coordinate corresponds to the distance from the center of the superconducting magnet.

The field from a superconducting magnet used for magnetic resonance is essentially axisymmetric. Moving along the axis away from the center of the magnet, one is facing very large gradients in the stray field, which dephases the signal extremely rapidly. While stray-field imaging techniques have used such gradients efficiently^13^, theoretical magnetostatics analysis shows that permanent magnets can be used to shape their profiles and reduce their strength^14^. Here, we used the simplest possible geometry of axially magnetized ring-magnets to show that the stray field gradient could be eliminated in multiple places along the symmetry axis of the superconducting magnet. The on-axis field dependence of the magnetic field produced by an axially magnetized ring can be written as:


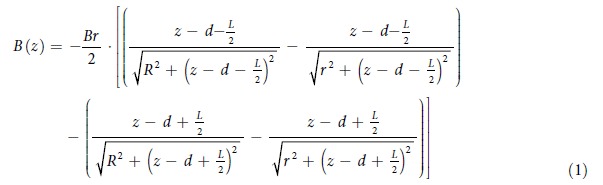


where *B*_*r*_ is the remanence of the chosen magnetic material and *d* is the position of the center of the magnetic ring along the z-axis. The volume of the magnetic ring depends on the thickness *L* along the z-axis, the outer radius *R*, and the inner radius *r*. The maximum value of the outer radius is limited by the size of the bore of the superconducting magnet. The lower limit for the inner radius is defined by the occupied area of the sample, or by the instrumentation that might be used at the sweet-spot. These parameters are shown in Figs 1(A) and 2(A).

The magnetic material could be rare earth alloys (NdFeB, or SmCo, or an optimized combination of them)^12^, with an ideally perfectly homogeneous magnetization inside its volume and reduced temperature dependence. Contrarily to “soft” magnetic materials, where their magnetization depends on the presence of strong extraneous magnetic fields, rare earth alloys exhibit “rigid” magnetization and are called “hard” magnets. However, in cases where the extraneous field exceeds a certain value, named coercivity, or in places where the geometry of magnetic piece is peculiar (e.g. corners, edges), one can observe deviations from an ideal uniform and rigid magnetization. Such effects are called demagnetization effects and in this paper will not be taken into account, since we will generate sweet spots in the range 0 to 1.5 Tesla. Even so, any remaining demagnetization effect will have a minor impact (a shift of the sweet spot position of less than 200 microns is numerically predicted). Furthermore, the rare earth permanent magnets have a dependence of ~1000 ppm/K and ~300 ppm/K for NdFeB and SmCo materials, respectively. Thus, the field drift due to temperature fluctuation can be solved by implementing a temperature compensation approach in magnet design^15^ or by applying an active feedback such as a lock in signal. These implementations at the low field generate sufficient stability for NMR experiments.

The precise knowledge of the magnetic field along the axis of the superconducting magnet is essential for the design of the corrective insert. Even if this work is numerical, we intended to perform the analysis with an experimentally measured magnetic field profile. We therefore measured all three components of the magnetic field inside an 11.7 Tesla Bruker Ultrashield, 89 mm free bore, magnet using a Senis 3-axis calibrated Hall probe (model 3MH3-20T) and an automated mapping tool adapted from a sample shuttling device^5^. The measurements were performed from a position below the center of the superconducting magnet to the open top of its Dewar, (the inner radius of the bore is 44.5 mm). The magnetic field was measured at 5490 positions along the symmetry axis, with a 0.2 mm step distance between two points. Sample results in the region of interest are shown in Figs 1(B) and 2(B) (red curves). A 12^th^ order polynomial function was fitted to the experimental magnetic field profile between 2.34 T and 0.06 T. The quality of the interpolation was tested with respect to the maximum order of the polynomial function, to reproduce the magnetic field profile with a precision better than 20 ppm. This polynomial function was used later in the optimization of the dimensions of the magnets.

## Results

We first propose to prove that three additional low-homogeneity centers can be easily achieved using simply three axially magnetized permanent magnet rings. The magnetized rings can be positioned with their magnetization opposed to, or along the stray field *B* of the superconducting magnet. Both options can be used to eliminate the gradient, but here we chose to use rings with parallel magnetization. This option has less demagnetization issues. We performed numerical calculations to optimize the dimensions of the rings, using Equation (1) and the polynomial function representing the field. We decided to fully cancel the first order linear gradient in three spots with magnetic fields equal to 1.0 T, 0.5 T and 0.3 T at distances of 41, 47, and 52 cm (respectively) from the center of the superconducting magnet. A non-linear minimization of the total magnets volume, under three constraints was performed in Maple. The target was a complete cancellation of the first order deviation of the magnetic field at these three spots. The inner radii for all pieces were fixed to *r* = 29 mm. The only reason behind this choice is that the final free bore can easily accommodate standard bore equipment (27 mm in radius) such as electrical shims, probes. The outer radii *R*_*i*_ were free for optimization, while their upper limit was set to *R*_*outer*_ = 44.5 mm. The thicknesses of the pieces were not constrained. The optimized dimensions of the permanent magnets are shown in Table 1. These magnets could easily be installed inside the 44.5 mm radius bore of our 11.7 T wide-bore magnet. Figure 1(B) shows the *B*_*z*_ component of the magnetic field of superconducting magnet alone and with the three permanent magnet rings. The three homogeneous spots define a “field ladder”. The homogeneity of the three spots was calculated to be less than 200 ppm peak to peak, inside a 2 mm diameter spherical volume (DSV), which would be sufficient for many applications.

Some applications require the design of a single spot of high homogeneity. We also demonstrate that one additional high-homogeneity center could be readily generated using two axially magnetized permanent magnet rings placed in an antiparallel configuration. Here the pair of rings produce a large magnetic field gradient as well as higher order zonal (i.e. axisymmetric) spherical harmonics^11^ that could be optimized to eliminate the zonal harmonics from the stray field of the superconducting magnet up to the 3^rd^ order. We decided to optimize the ring dimensions to obtain a single spot where first, second and third order zonal harmonics were exactly canceled at the field 0.719 T. We used a non-linear optimization under constraints programmed in Maple. The inner radii for both pieces were fixed to *r* = 29 mm. The outer radii R_i_ were free for optimization, while their upper limit was set to *R*_*outer*_ = 44.5 mm. The thicknesses of the pieces were not constrained. The optimized dimensions of the two magnet rings are shown in Table 2. Again, such magnets could easily fit inside the 44.5 mm radius bore of our 11.7 Tesla wide-bore magnet. Figure 2(B) presents the B_z_ component of the magnetic field of the superconducting magnet with and without the antiparallel magnetic pair ring. The magnetic field inhomogeneities were less than 74 ppm peak to peak, inside a 10 mm DSV at homogeneous spot. This should be practical for most experiments and close to the typical homogeneity required by simple high-resolution spectroscopy experiments.

## Discussion

The polynomial order of gradient compensation determines the volume of the sweet spot, as can be observed by comparing Figs 1(B) and 2(B). For some applications, a simple first-degree compensation is sufficient, especially if the sample volume is limited (length of a few mm) for instance in dynamic nuclear polarization (DNP) applications. In other cases, where the length of the sample is required to be around 1 to 2 cm, or alternatively, where some applications require a very homogeneous sweet spot (like NMR spectroscopy), high-order compensation becomes essential. In that case, a larger number of magnet rings and components will have to be used and the volume of the required magnetic materials will become important.

High coercivity gives our design approach a clear advantage compared to ferromagnetic shims, since the rigid magnetization provides the freedom to design magnetic structures with antiparallel magnetizations (see Fig. 2(A)), having partly compensated net dipole moment. This option is particularly appealing for the development of highly compensated sweet-spots magnetic inserts, since the overall force with the superconducting magnet can be greatly reduced. We are currently working on such designs but their description and validation is beyond the scope of the current publication. Another advantage of our approach, resides in our ability to work in regions where a ferromagnetic material could not be properly magnetized. For example, in the range of high (0.5 T and above) fields its remanence is limited at full saturation. Preliminary simulations (not presented here) using the RADIA code^16^ show that the free bore diameter is greatly reduced when using ferromagnetic inserts, and it becomes much less than 54 mm, which is the standard bore size for high field NMR experiments. Alternatively, at low fields (below 0.1 T) ferromagnetic materials can be imperfectly saturated and their field generation properties poorly controlled. To the best of our knowledge only a 0.3 T ferromagnetic shim insert has been designed (see [9]).

Demagnetization effects limit, however, the applicability of our approach. These will be particularly important when the aim is to achieve high field sweet spots when using pieces having an antiparallel magnetization with respect to the main magnetic field. Although modern rare-earth magnets have high coercivity fields^12^, they rarely exceed 1.5–2 T without a massive and irreversible drop in magnetization. Thus one has to take carefully into account these effects using a numerical simulation tool beyond the simple numerical expressions given in our paper.

The theoretical calculations presented here were carried out under realistic conditions. The values of the remanence used in the calculations are achievable commercially, as well as the dimensions and tolerances. High precision (+/−25microns) grinding and magnetizing such permanent magnet rings is fairly standard and can be performed by some specialized companies at reasonable costs. One has to be however attentive while working with such strong permanent magnets to avoid hazards that can be extremely dangerous for operators and for the magnets. Thus, we insist that extreme caution is required upon building and adapting such inserts inside superconducting magnets.

Furthermore, it will be necessary to fine-adjust experimentally the position of each ring with respect to the center of the magnet in order to achieve perfect compensation. High-homogeneity (sub-ppm) centers will be achieved with permanent magnet rings when combined to electrical shims and signal lock tracking. Such high degree of field compensation could be used to provide coherent spin manipulation and high-resolution spectroscopy also at low magnetic fields and allow one to record low field NMR spectra without the need of a second magnet. Furthermore, low field spectra could be detected indirectly in high field by sample shuttling similarly to zero-field NMR^17^ or remote detection NMR^18^. Such correlated high-field and low-field NMR spectroscopy and relaxometry could open new directions in NMR and MRI methodology.

Notice that other magnetization orientations could be used to offer better possibilities for field correction. For example, rings with radial magnetization produce different zonal harmonics and have different efficiency per unit volume when compared with axially magnetized rings. Another advantage of radial orientations comes from its higher resistance to demagnetization effects, due to the very strong anisotropy of the easy magnetization axis.

## Conclusions

In this communication, we introduce a simple and low-cost, practical approach in order to produce multiple homogenous volumes inside the bore and along the center axis of a superconducting magnet. The theoretical work presented here demonstrated that a combination of elementary permanent magnet inserts could be built to compensate the gradient in realistic magnetic field profiles, and capably installed inside the bore of the superconducting magnet. More complicated structures, combining various orientations of magnetization, could be designed and built for achieving high-resolution NMR at the low-field centers as well, extending significantly the potential of NMR spin excitation, manipulation and detection between multiple magnetic field centers. Work in these directions is currently undertaken in our laboratory.

## Additional Information

**How to cite this article**: Chou, C.Y. *et al*. Simple method for the generation of multiple homogeneous field volumes inside the bore of superconducting magnets. *Sci. Rep*. **5**, 12200; doi: 10.1038/srep12200 (2015).

## Figures and Tables

**Table 1:** Table with the geometric parameters of the three rings after the first order field optimization.

	*z* position from magnetic center (cm)	*R*_*i*_ (mm)	Thickness *L* (mm)	Final Field B (T)
Ring 1	41.00	43.50	14.02	0.986
Ring 2	47.24	38.30	8.87	0.525
Ring 3	52.28	34.42	7.06	0.328

The inner radii for all three rings were set to 29.00 mm, and the remanence *B*_*r*_ was fixed to 1.3 T. The last column informs about the final value of the magnetic field at the z positions where the linear gradient was canceled.

**Table 2:** Table with the geometric parameters of the two rings after the third order field optimization.

	*z* position from magnetic center (cm)	*R*_*i*_ (mm)	Thickness *L* (mm)	Gap *d* (mm)	Final Field *B* (T)
Ring 1	41.00	40.48	35.57	27.21	0.719
Ring 2	46.42	39.42	18.48		

The inner radii for the two rings were set to 29.00 mm, and the remanence *B*_*r*_ was fixed to 1.3 T. The optimized gap *d* was defined as the distance between the two centers of the rings. The last column informs about the final value of the magnetic field at the z position of the sweet spot.
